# Supplementary material for: Pulmonary function and comparative SARS-CoV-2 RBD-specific IgG antibody response among the COVID-19 recovered group
Source: PLoS One. 2025 Jul 11;20(7):e0318959. doi: 10.1371/journal.pone.0318959 (PMC12250562; doi:10.1371/journal.pone.0318959)
Supplement: S2 File — PFT parameters including forced vital capacity (FVC), forced expiratory volume in 1 second (FEV1), FEV1/FVC, peak expiratory flow (PEF), maximal expiratory flow (MEF), forced expiratory flow (FEF) with pre and post bronchodilation %predicted scores both before and after vaccination among the mild, moderate, severe and control study groups. %predicted score shown in median value with interquartile range (IQR). (DOCX) [file pone.0318959.s002.docx]

| **Spirometry test parameters** | **Mild (n=15)** | | **Moderate (n=6)** | | **Severe (n=8)** | | **Control (n=64)** | |
| --- | --- | --- | --- | --- | --- | --- | --- | --- |
|  | **Pre-Vac** | **Post-Vac** | **Pre-Vac** | **Post-Vac** | **Pre-Vac** | **Post-Vac** | **Pre-Vac** | **Post-Vac** |
| **FVC (%predicted), median (IQR)** |  | | | | | | | |
| prebronchodilator | 73 (71, 78) | 77 (66, 82) | 66 (51, 77) | 84 (79, 92) | 67 (65, 81) | 70 (66, 79) | 80 (75, 89) | 81 (71, 89) |
| postbronchodilator | 74 (68, 77) | 63 (63, 63) | 56 (47, 64) | NA (NA, NA) | 60 (55, 65) | 62 (62, 62) | 71 (67, 75) | 65 (60, 72) |
| **FEV1 (%predicted), median (IQR)** |  | | | | | | | |
| prebronchodilator | 77 (74, 83) | 77 (70, 83) | 70 (56, 80) | 88 (82, 96) | 75 (68, 89) | 68 (66, 81) | 82 (76, 90) | 80 (72, 89) |
| postbronchodilator | 77 (75, 81) | 66 (66, 66) | 62 (53, 67) | NA (NA, NA) | 65 (63, 67) | 63 (63, 63) | 73 (70, 77) | 69 (64, 75) |
| **FEV1/FVC (%predicted), median (IQR)** |  | | | | | | | |
| prebronchodilator | 109 (104, 112) | 106 (102, 108) | 114 (112, 116) | 110 (105, 114) | 112 (110, 119) | 107 (105, 109) | 108 (103, 111) | 107 (101, 111) |
| postbronchodilator | 110 (107, 114) | 108 (108, 108) | 116 (113, 119) | NA (NA, NA) | 116 (112, 120) | 106 (106, 106) | 107 (100, 115) | 109 (107, 114) |
| **PEF L/s (%predicted), median (IQR)** |  | | | | | | | |
| prebronchodilator | 92 (84, 99) | 97 (87, 112) | 75 (52, 99) | 90 (73, 108) | 77 (70, 93) | 85 (70, 92) | 91 (80, 101) | 91 (87, 103) |
| postbronchodilator | 93 (86, 100) | 66 (66, 66) | 60 (50, 79) | NA (NA, NA) | 91 (68, 112) | 75 (75, 75) | 83 (78, 93) | 91 (78, 97) |
| **MEF 25% L/s (%predicted), median (IQR)** |  | | | | | | | |
| prebronchodilator | 72 (57, 94) | 74 (62, 80) | 84 (74, 92) | 74 (60, 92) | 71 (71, 87) | 58 (58, 79) | 78 (66, 104) | 71 (60, 90) |
| postbronchodilator | 80 (64, 90) | 71 (71, 71) | 77 (71, 89) | NA (NA, NA) | 72 (65, 103) | 57 (57, 57) | 72 (57, 104) | 65 (48, 80) |
| **MEF 50% L/s (%predicted), median (IQR)** |  | | | | | | | |
| prebronchodilator | 82 (71, 105) | 76 (65, 87) | 92 (74, 101) | 84 (65, 93) | 75 (74, 84) | 85 (73, 93) | 85 (73, 100) | 80 (65, 95) |
| postbronchodilator | 86 (71, 98) | 73 (73, 73) | 76 (57, 94) | NA (NA, NA) | 73 (65, 97) | 66 (66, 66) | 75 (59, 91) | 76 (59, 80) |
| **MEF 75% L/s (%predicted), median (IQR)** |  | | | | | | | |
| prebronchodilator | 91 (75, 100) | 88 (74, 111) | 65 (53, 88) | 95 (69, 117) | 73 (66, 96) | 71 (68, 83) | 89 (76, 100) | 86 (71, 98) |
| postbronchodilator | 93 (83, 98) | 71 (71, 71) | 65 (54, 82) | NA (NA, NA) | 94 (70, 119) | 50 (50, 50) | 79 (73, 87) | 82 (75, 97) |
| **FEF L/s, (% predicted), median (IQR)** |  | | | | | | | |
| prebronchodilator | 77 (69, 99) | 71 (66, 80) | 84 (74, 92) | 85 (70, 92) | 71 (71, 87) | 69 (62, 83) | 79 (67, 97) | 74 (62, 89) |
| postbronchodilator | 83 (69, 89) | 71 (71, 71) | 76 (62, 90) | NA (NA, NA) | 76 (63, 103) | 66 (66, 66) | 68 (57, 96) | 63 (56, 78) |
